# Anemonefish prioritize mutualistic partner over social group during a marine heatwave

**DOI:** 10.1098/rsos.261623

**Published:** 2026-11-11

**Authors:** Fletcher John Noble, Melissa Versteeg, Kirstin Gaffney, Alexandra Grace Adams, Anoushka Pinto, Eve Stephenson-Archibald, Laura Sullivan, Theresa Rueger

**Affiliations:** ^1^ Dove Marine Laboratory, School of Natural and Environmental Science, Newcastle University, Newcastle upon Tyne, UK; ^2^ Marine Eco-Evo-Devo Unit, Okinawa Institute of Science and Technology Research Laboratories, Kunigami, Japan

**Keywords:** climate change, coral reefs, environmental change, symbiosis, Papua New Guinea, maintenance behaviour, defence behaviour, social behaviour, bleaching, Zooxanthellae

## Abstract

Changing their behaviour is animals' first line of defence in response to environmental disturbance. However, changes in behaviour towards social group members and mutualistic partners can also lead to reduced fitness and resilience. Here, we use clown anemonefish, *Amphiprion percula*, to understand how disturbances alter social and mutualistic behaviours directly and indirectly via degraded mutualistic partners. We measured fish behaviour from video recordings made during a marine heatwave in Kimbe Bay, Papua New Guinea. Since some of the hosting anemones bleached, lost symbiotic algae, and others did not, we were able to estimate the effects of heat stress separate from the indirect effects through stressed mutualistic partners. Neither stressor significantly impacted activity levels or interactions with the mutualistic partner, which made up a high proportion of behaviours throughout. However, bleaching did increase aggression between group members and defensive behaviours. Our results provide an example of behavioural plasticity in response to environmental disturbance where different behaviours were adjusted to the stressor itself or to the changing habitat. We show that some species will prioritize interactions with mutualistic partners, while their social cohesion is weakened.

## 1. Introduction

The first response of animals to environmental change is often through changes in behaviour [1]. Behavioural responses determine how mutualistic partnerships, such as with habitat building plants or animals, are impacted by environmental change and whether such partners contribute to resilience or exacerbate the impact of change. For example, in the ant–aphid mutualism, temperature increases cause a cascade in behavioural responses by increasing aphid activity, which in turns triggers increased aggression by ants towards them [2,3]. In times of low protein availability, there is even a switch from mutualism to predation as ants consume aphids to make up the deficit [2–4]. Other mutualisms are reported to be disrupted or altered owing to environmental change, including pollinator–plant, seed distributors–plant and cleaner–client relationships [5–12]. Changes in mutualisms can have wide ranging effects on food webs and ecosystems [13,14] and can even be drivers of co-extinctions when relationships fully collapse [15].

Environmental change can also impact animal social behaviours. Many animals live within tight social groups or hierarchies and their behaviours within these groups can have direct impacts on their fitness, including, foraging ability or breeding success. For example, habitat fragmentation can split groups of wolves, *Canis* spp., changing foraging behaviour [16] or limiting mate choice resulting in inbreeding [17], impacting breeding success and the genetic health of the population. In cichlids (*Cichlidae*), elevated temperature is associated with increased aggression and unstable dominance hierarchies [18,19]. Temperature increases can lead to flipped hierarchies in cockroaches (*Nauphoeta cinerea*), where dominants become submissive and subordinates become aggressive [20]. Changes in social behaviour, like changes in mutualistic relationships, can have fast impacts on individual fitness with consequences for population persistence [21,22].

In marine environments, much is still unknown about behavioural plasticity in response to environmental change, even though increasingly frequent marine heatwaves threaten populations across many marine ecosystems [23]. Marine heatwaves are defined as discrete, prolonged and anomalous warm water events which persist for 5 days or more [24]. These increases in temperature are a source of heat stress for many species resulting in decreased survival [25,26], as well as sub-lethal effects on growth [27–29], and fecundity [30]. While marine heatwaves occur globally, their impacts are most dramatically felt on species already on the edge of their thermal tolerance owing to the climate zone they inhabit and their lack of mobility [31–35]. Tropical shallow water species therefore are some of the most impacted by marine heatwaves [36–38]. On tropical coral reefs, one of the clearest consequences of prolonged heat stress on habitat building cnidaria, corals and anemones, is bleaching, the ejection or digestion of symbiotic photosynthetic algae which are important for energy acquisition [39,40]. Global bleaching events are becoming more frequent [41–44], threatening both the habitats cnidaria create and the species which inhabit them [45].

Coral reef fishes depend on the habitat created by corals and other habitat builders for food and shelter [46–49]. During coral bleaching events, reef fishes change their foraging behaviour [35,50–53], predator–prey interactions [54–56] and aggression levels [55,57–59]. Rising water temperatures also have direct impacts on fish thermo-regulatory behaviour, seeking shelter and cooler environments [60,61]. Fish exposed to high temperatures increase or decrease activity levels, depending on the species and life stage [52,62,63], and increase or decrease swimming speed [62–64]. Owing to the difficulties of measuring fish behaviour *in situ* for highly mobile reef species, most studies to date are confined to either laboratories or short-term monitoring of a limited set of behaviours and the effects of rising temperatures cannot be disentangled from the effect of habitat bleaching.

Anemonefishes provide a tractable system to understand behavioural plasticity in response to marine heatwaves, as they are strongly site attached owing to their obligate associations with mutualistic anemone hosts. Anemones and anemonefishes provide each other with defence against predators and act as a source of additional nutrients [65–67], with anemonefishes actively defending, aerating and cleaning their hosts [68,69]. Occupying one or several species of host anemone, anemonefish live in pairs or groups characterized by strict size hierarchies, which are maintained through aggression, submission and cooperative behaviours [69–71]. The condition of the host anemones and occupying anemonefish are closely linked. For example, when anemones bleach, anemonefish experience raised metabolic rates, reduced growth rates, reduced feeding rates and reduced fecundity [66,67,72]. Similarly, juveniles exposed to high temperatures in laboratory studies had altered foraging activity, metabolic rates, swimming speeds and growth rates [73,74]. However, measures of behavioural responses to bleaching or elevated temperature have so far been limited. Studies using artificially bleached anemones, without the accompanying temperature rise, found that juvenile *Amphiprion chrysopterus* were less active [75] and adult *A. akindynos* had altered predator responses [76]. The only study, to our knowledge, which followed anemonefish through a naturally occurring marine heatwave found that anemone bleaching impacted fish fecundity more than the spike in temperature alone [66], highlighting the importance of disentangling direct effects of temperature from the indirect effects experienced through a degraded mutualistic host.

Our study aimed to disentangle the effects of heat stress and cnidarian host bleaching on social and mutualistic behaviours in site attached coral reef fishes. We recorded the behaviour of 69 clown anemonefish, *A. percula*, across 23 social groups in Kimbe Bay, Papua New Guinea, during a marine heatwave that caused bleaching in some but not all host anemones. We recorded behaviour at the start of the heatwave when temperatures were at their highest, but no bleaching had occurred, and again at the end when temperatures normalized but hosts were bleached. Our first objective was to understand whether anemonefish change their behaviour patterns when their host bleaches. We predicted that as the heatwave progresses, activity levels of the anemonefish change more in fish whose anemone host bleaches compared to fish in unbleached hosts [55,59,66]. Our second objective was to understand how heat stress and bleaching affect interactions with the anemone host and the mutualistic services anemonefish provide. We predicted that fish will spend more time hiding at higher temperatures, but this effect will be greater in bleached anemones. Previous work has shown that fish adjust their behaviour to seek more shelter and thermo-regulate as temperatures rise [60,61], while bleaching can reduce activity and has greater impacts on the fish than temperature alone [66,75,76]. Fishes in bleached hosts are more conspicuous and may spend more time sheltering from predators [54,77,78]. Our third objective was to understand the impact of heat stress and bleaching on social behaviours within anemonefish social groups. We predicted increased levels of aggression by individuals in bleached anemones and less neutral interactions as increased levels of stress can result in break downs of social cohesion, as seen in other damselfishes which compete for space within degraded habitat [57,79,80].

## 2. Methods

### 2.1. Field site and study species

This study took place on seven inshore coral reefs in Kimbe Bay, Papua New Guinea (5°30′ S, 150°05′ E) with researchers based at Mahonia Na Dari Conservation and Research Centre in West New Britain province. Twenty-three social groups of *A. percula* on individual magnificent sea anemones, *Radianthus magnifica*, were monitored through a marine heatwave from its start in March 2023 to the first week of July. We followed the definition of marine heatwaves by Hobday *et al.* [24], discrete, prolonged and anomalous warm water events with temperatures reaching more than the 30-year 90th percentile. Water temperatures were greatest during the first monitoring (time point 1) and then fell during the second monitoring (time point 2; figure 1A) returning to normal levels, as compared to previous work by Galbraith *et al.* which reported an annual mean temperature of 30.82°C [81]. Groups were identified using plastic tags attached to the reef substrate within 1 m of the anemone. In the beginning of the study, there were 11 groups of 2 individuals and 12 groups of 3 individuals. One group lost one individual, and one group gained one individual through recruitment of a subordinate. Within each group the rank of each fish was determined using body length which is the basis for the strict social hierarchy [82,83]. Fish were caught in hand nets, measured for total length to the nearest millimetre using callipers and photographed for later identification at the end of each time point after all videos were recorded [29,69]. Fish were caught at the end of the study period, at the end of the lunar month. The area of the anemone oral disc has been shown to limit group size [84] and may therefore impact social behaviour as space available is constrained. To control for this, anemone size was recorded as the area of an oval, measured with tailor's tape at each of the two recordings.

**Figure 1.**
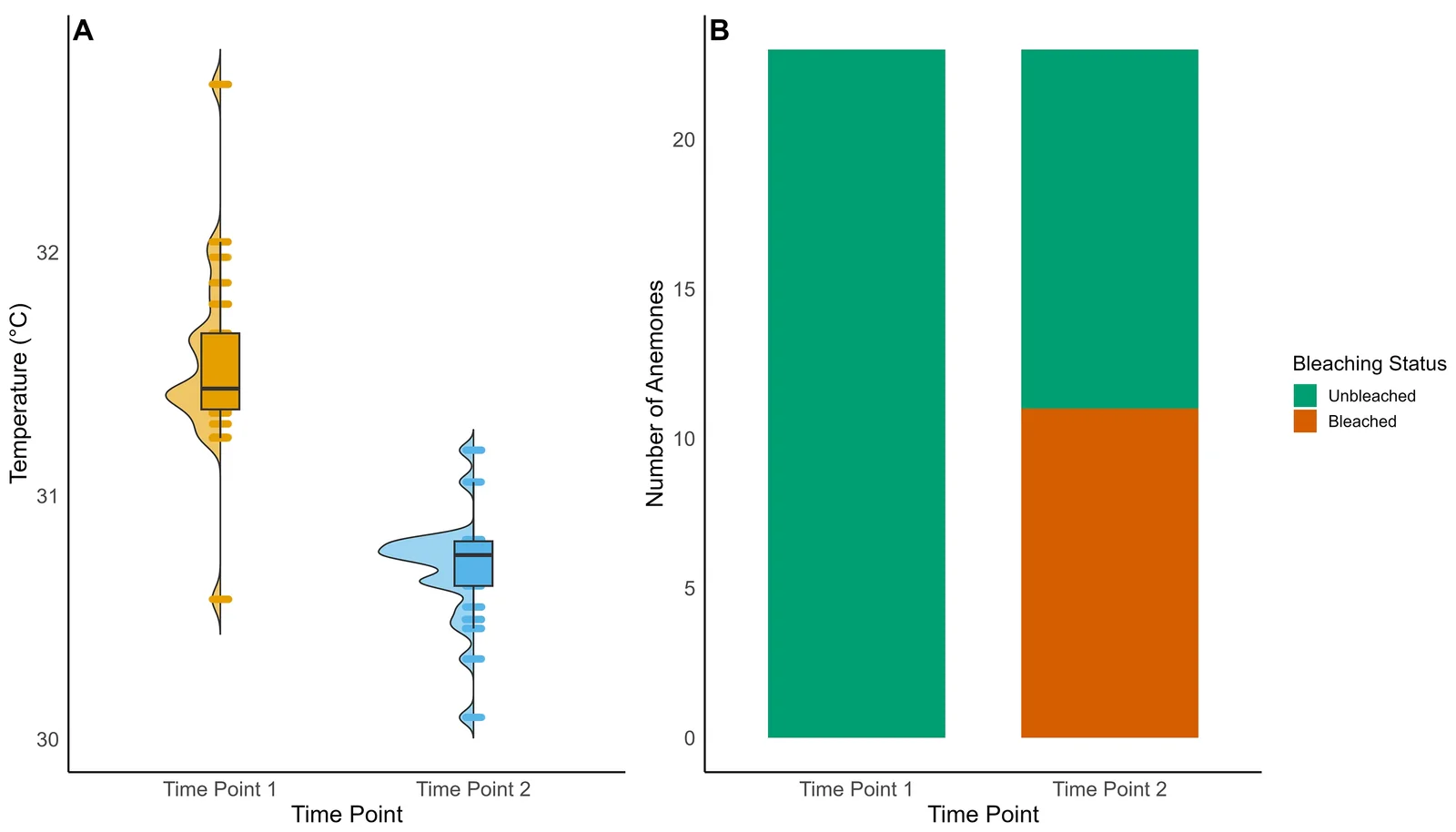
Environmental and anemone host condition for 23 groups of *A. percula* in Kimbe Bay, Papua New Guinea, at each of two monitoring time points. (A) Temperature measured at each group during time point 1 (March–April 2023) and time point 2 (May–July 2023) of the study, showing 25–75% quantile box and median line, with 0–25% and 75–100% quantile whiskers. The ribbon represents the density plot of all individual temperature measurements. (B) Number of bleached anemone hosts (*R. magnifica*) for all groups during each time point.

### 2.2. Behaviour recording

Each anemonefish social group was video recorded twice throughout the study period, once at the beginning of the marine heatwave prior to bleaching (time point 1, March–April) and once later in the heatwave once bleaching occurred (time point 2, May–July). This allowed us to compare behaviours at levels of high thermal stress and no bleaching (time point 1) and reduced thermal stress but bleached anemone (i.e. degraded habitat; time point 2). Before recording, non-breeding status of anemonefish groups was confirmed to avoid behavioural changes from parental care [85,86]. We also checked that anemones had extended their tentacles before recording, as not to limit space available to the fish.

To collect video footage for behavioural observations of anemonefish, GoPro Hero9 action cameras were deployed on tripods 1.5–2 m away from each anemone for 20 minutes. Cameras were deployed using SCUBA and left to record without diver presence to minimize disturbance. Habituation to cameras occurs within 2–7 minutes for coral reef fishes [87].

### 2.3. Assessment of temperature and bleaching status

Bleached anemones were recognized by trained divers using the CoralWatch Coral Reef Health Chart [41]. Anemones were judged to have been bleached when their colour matched the lowest value for brightness and saturation on the chart no matter the hue (e.g. B1, C1, D1, E1). Bleaching was only present during time point 2 (figure 1B). All mention of bleaching in anemones in this study is referring to their status during time point 2.

To assess heat stress experienced by resident fish at microhabitat level, temperature was recorded at each anemone every 4–6 days throughout the heatwave using a HOBO MX2202 temperature logger placed within 1 m of the target anemone. The logger was set to record temperature at 2-minute intervals, left for up to 10 minutes, and the daily average extracted. These data allowed for temperature to be averaged for each time point [87–89].

### 2.4. Behaviour scoring

The videos from each time point were scored using the software BORIS [88]. From the total 20 minutes of video recorded, 15 minutes of footage was scored for each fish in the social group. The first 5 minutes of footage was discarded to allow for the fish to habituate to the camera and for scuba divers to depart. Behaviours were categorized into ‘social’ and ‘mutualistic’. Social behaviours were divided into three further categories, aggressive, submissive and neutral, while mutualistic behaviours were further divided into defence and maintenance [71,89] (supplementary material, table S1).

Individuals were recognized based on their colour markings (unique patterns of their orange, white and black colour bands) along with size differences owing to the strict body length ratio which is the basis of the social group hierarchy. These identification methods are common throughout this genus [70,82]. The amount of time the fish was not visible during the footage was also recorded; this was used to statistically offset the number of behaviours by sampling effort (i.e. the number of minutes the fish was visible) using the offset function in *glmmTMB* package [90]. Time visible was utilized as a general measure of activity by *A. percula.*

### 2.5. Statistical modelling

All statistical analysis was done in R, version 4.4.0 [91].

To test how levels of overall activity changed in bleached and unbleached anemones during the heatwave, we used an identity linked gaussian linear mixed model. Proportion of time visible was used as the response variable. The predictors included a two-way interaction between the time point (binary: time point 1/2) and bleaching status of the anemone during time point 2 (binary: bleached/unbleached). Rank (place in social group hierarchy, categorical: rank 1, 2, 3), behaviours per second (rate of all behaviours during time visible, continuous), social group size (number of fish inhabiting anemone, integer), microhabitat temperature (degrees Celsius, continuous) and anemone size (area of oral disc, continuous) were used as co-variates. Group ID and fish ID were used as random variables to account for repeated measures and non-independence.

To test how behaviour patterns change in *A. percula* in response to heat stress and anemone host bleaching, we calculated five separate models examining how the rate of behaviours changed for each behavioural category (maintenance, defence, aggressive, neutral and submissive). We used the count of each behavioural category offset by log time visible (rate, continuous) as the response variable, and as predictors included an interaction between time point (binary: time point 1/2) and bleaching status of the anemone during time point 2 (binary: bleached/unbleached). Social group size, anemone size, microhabitat temperature and social rank of the focal fish were co-variates and group ID and fish ID were used as random variables to account for repeat measures and non-independence. Fish ID resulted in a singularity in models for neutral and submissive behaviours, indicating fish ID accounted for near zero variance. A sensitivity test done on one of the models (neutral) (supplementary material, table S2) confirmed that the inclusion of fish ID did not impact the model, and we consequently retained it in all models to keep model structure consistent. This follows examples and recommendations from other behavioural studies where random effects initially trigger singularities and were retained [92,93]. All models were fitted with a negative binomial type 1 family link fitted with the *glmmTMB* package [90,92].

Model choice was assessed by simplifying the model and using log likelihood tests to test for significant changes in the model and removing the covariate. The fit of the individual models was analysed using the *performance* and DHARMa packages [94,95]. Tests of fit included: posterior predictive check; linearity; homogeneity of variance; collinearity; normality of residuals and normality of random effects. Post-hoc analysis was done to compare behaviours during time point 1 and time point 2, and to compare bleached and unbleached anemones. This was completed using the *emmeans* package and the standard Tukey correction [96]. To account for the large differences in range of values in continuous variables, microhabitat temperature and anemone size were scaled.

## 3. Results

### 3.1. Activity levels

The activity levels of anemonefish (time visible as a proportion of observation time) remained unchanged throughout the study period, being unaffected by time point (*β* = −0.0445, CI = −0.229 to 0.140, *t*-value = −0.480, *p*-value = 0.633), bleaching status (*β* = −0.048, CI = −0.189 to 0.094, *t*-value = −0.667, *p*-value = 0.510) or the interaction of the two (*β* = −0.027, CI = −0.173 to 0.118, *t*-value = −0.375, *p*-value = 0.708). Fish in unbleached anemones experienced a small decrease in activity at time point 2, falling by 6.7% on average (emmeans contrast: CI = −0.223 to 0.144, *t*-ratio = −0.471, *p*-value = 0.639), while there was a greater reduction in activity in bleached anemones, 11.7% (CI = −0.201 to 0.057, *t*-ratio = −1.113, *p*-value = 0.269), though the difference was non-significant.

The smallest anemonefish in the group, rank 3, was significantly less active than the breeding pair, being visible on average 35.1% and 29.1% less than rank 1 and rank 2, respectively (rank 3–rank 1: CI = −0.367 to −0.128, *t*-value = −5.057, *p*-value = < 0.001; rank 3–rank 2: CI = −0.325 to −0.086, *t*-ratio = −4.178, *p*-value = < 0.001), while rank 2 was only 6.0% less active than rank 1 (CI = −0.150 to 0.065, *t*-value = −0.958, *p*-value = 0.607). Fish which performed more behaviours per second spent more time inactive overall (*β* = −2.705, CI = −4.886 to −0.525, *t*-value = −2.459, *p*-value = 0.016). Microhabitat temperature, anemone size and social group size were all non-significant (supplementary material, table S3.1). Fixed effects accounted for 25.4% of variance in the model, while fixed and random effects together accounted for 50.9% (marginal *R*^2^ = 0.254, conditional *R*^2^ = 0.509; figure 2).

**Figure 2.**
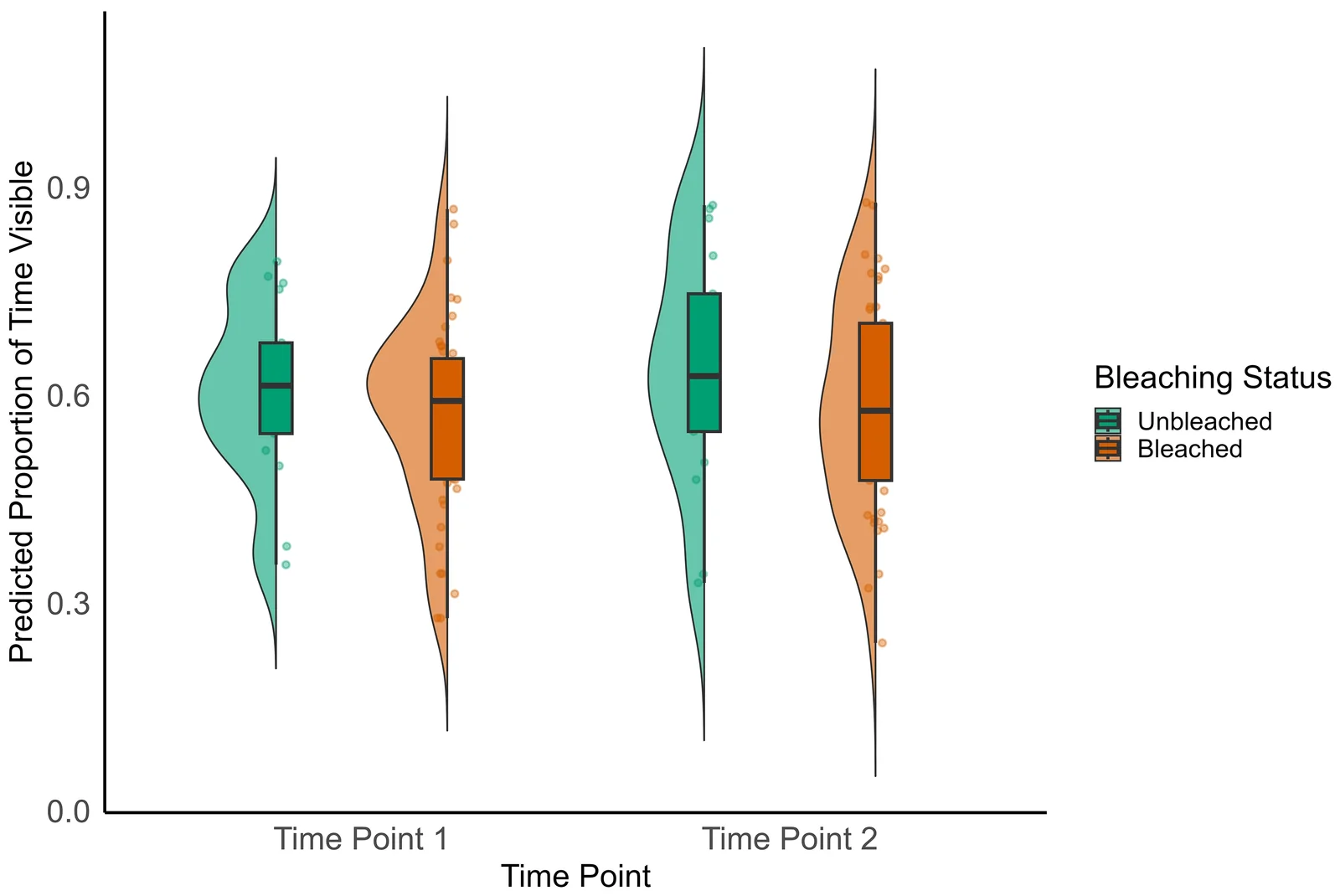
Activity levels of *A. percula* throughout the marine heatwave. Predicted proportion of time visible both at the height of the heatwave (time point 1) and at the end of the heatwave (time point 2) for bleached (*n* = 11, orange) and unbleached (*n* = 12, green) anemones. Points represent the predicted proportion of time visible for each anemone. Box plot shows 25–75% quantiles of distribution with the middle bar the median value and whiskers the 0–25% and 75–100% quantiles. Ribbon shows the full distribution of predicted points of the model.

### 3.2. Social behaviours

#### 3.2.1. Aggressive

Aggression among group members was impacted by bleaching status and changed through the study period (figure 3A). The rate of aggressive behaviour increased in bleached anemones by 215.0% between time points 1 and 2 (ratio = 3.150, CI = 1.465–6.770, *z*-ratio = 2.937, *p*-value = 0.003) while in unbleached anemones it decreased by 60.9% (ratio = 0.391, CI = 0.061–2.520, *z*-ratio = −0.988, *p*-value = 0.323). At time point 2, fish in unbleached anemones performed 86.1% less aggressive behaviours than those in bleached anemones (ratio = 0.139, CI = 0.016–1.240, *z*-ratio = −1.767, *p*-value = 0.077), at time point 1, there was no significant difference (ratio = 1.118, CI = 0.180–6.920, *z*-ratio = 0.120, *p*-value = 0.905).

**Figure 3.**
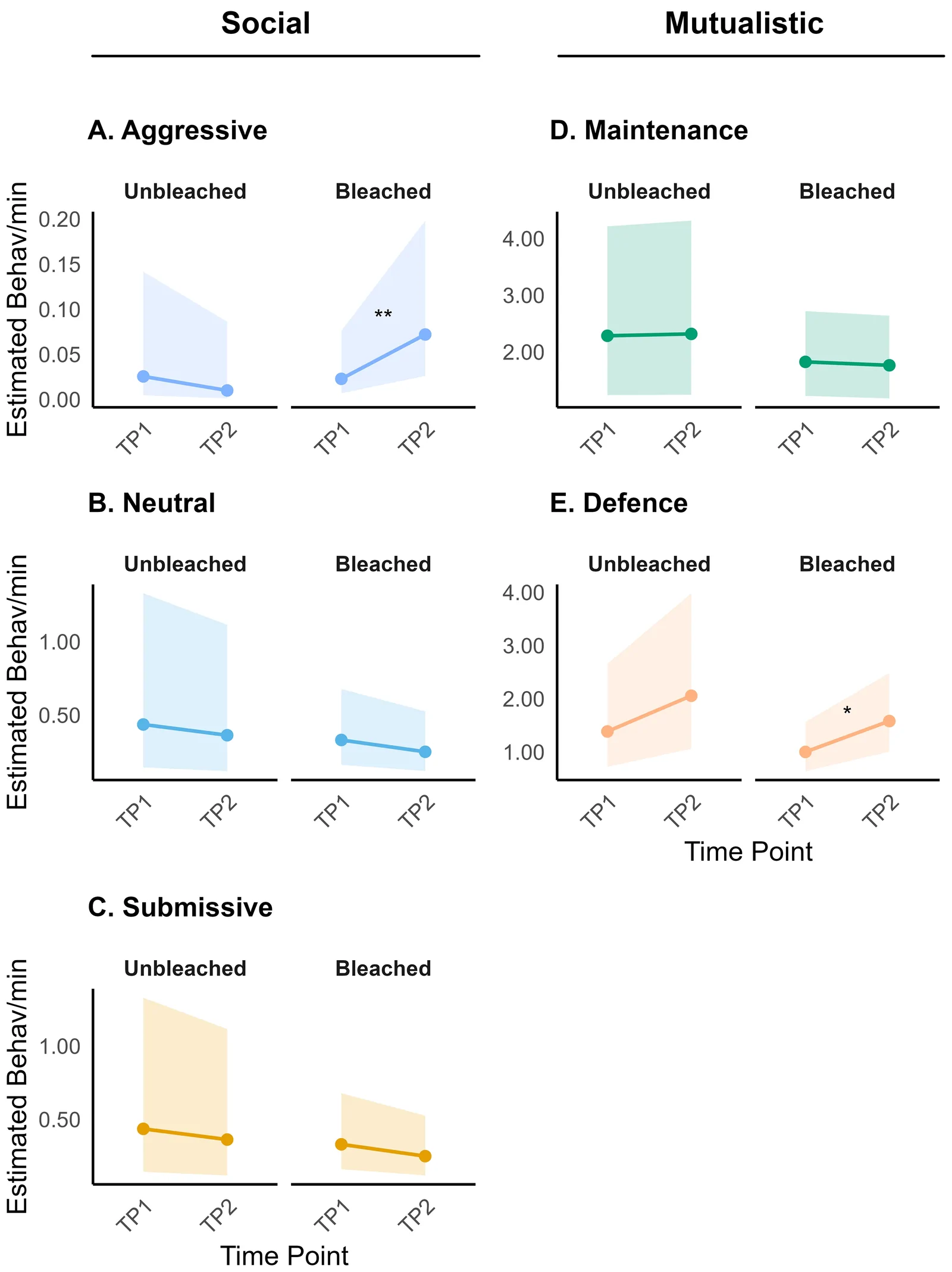
Change in rates of estimated displays of behaviour (behaviours per minute) from each behavioural category by *A. percula* in bleached (*n* = 11) and unbleached (*n* = 12) anemones at time point 1, at the height of the heatwave, and 2, at the end of the heatwave. The shaded ribbons represent 95% confidence intervals, while each point represents the mean at each time point. Estimated behaviours per minute are extracted from the emmeans contrasts of categorical behaviour models, which calculates the rates of each behaviour by offsetting the count of each behaviour by the log of time observed to get behaviours per second.

The smallest fish (rank 3) displayed significantly fewer aggressive behaviours than both rank 1 (67.4%, emmeans contrast: ratio = 0.326, CI = 0.127–0.839, *z*-ratio = −2.779, *p*-value = 0.015) and rank 2 (68.2%, ratio = 0.318, CI = 0.122–0.833, *z*-ratio = −2.789, *p*-value = 0.015). There was no difference in rates of aggression between rank 1 and rank 2 (ratio = 1.024, CI = 0.550–1.909, *z*-ratio = 0.091, *p*-value = 0.996). All other covariates had no significant impact on the rate of aggressive behaviour, including microhabitat temperature (supplementary material, table S4.1). Fixed effects alone explained 8.3% of the variance while fixed and random explained 34.3% (marginal *R*^2^ = 0.083, conditional *R*^2^ = 0.343).

#### 3.2.2. Neutral

Anemonefishes performed neutral behaviours at similar rates throughout the heatwave in both bleached and unbleached anemones (figure 3B). The rate of neutral behaviours in unbleached anemones decreased on average by 16.8% at time point 2 (emmeans contrast: ratio = 0.832, CI = 0.411–1.680, *z*-ratio = −0.512, *p*-value = 0.609) and decreased by 24.4% in bleached anemones (ratio = 0.756, CI = 0.463–1.230, *z*-ratio = −1.121, *p*-value = 0.262). While the difference in neutral behaviours widen from 32.0% greater in unbleached anemones compared to bleached at time point 1, to 45.3% at time point 2. These differences were non-significant (time point 1: ratio = 1.320, CI = 0.392–4.450, *z*-ratio = 0.448, *p*-value = 0.654; time point 2: ratio = 1.453, CI = 0.427–4.940, *z*-ratio = 0.598, *p*-value = 0.550).

No covariates had a significant impact on the rate of neutral behaviour (supplementary material, table S5.1). Rank 2 showed 32.8% more neutral interactions than rank 1 (emmeans contrast: ratio = 1.328, CI = 1.001–1.760, *z*-ratio = 2.354, *p*-value 0.049) but only 6.5% difference with rank 3 (ratio = 0.935, CI = 0.676–1.290, *z*-ratio = −0.490, *p*-value = 0.876). Similarly, there was a 24.1% difference between rank 3 and rank 1 which was not significant (ratio = 1.241, CI = 0.875–1.760, *z*-ratio = 1.449, *p*-value = 0.316). Fixed effects alone accounted for 3.7% of the variance while fixed and random effects accounted for 27.3% of the variance (marginal *R*^2^ = 0.037, conditional *R*^2^ = 0.273).

#### 3.2.3. Submissive

There was a lot of variance in the rate of submissive behaviours and no clear impact of anemone bleaching or the time period could be determined (figure 3C). The rate of submissive behaviour in anemonefish decreased on average by 56.5% in unbleached anemones from time point 1 to time point 2, while in bleached anemones it decreased by 69.4%, however, both changes were not significant (emmeans contrast: unbleached: ratio = 0.435, CI = 0.073–2.600, *z*-ratio = −0.911, *p*-value = 0.362; bleached: ratio = 0.306, CI = 0.077–1.220, *z*-ratio = −1.682, *p*-value = 0.093). When comparing the rates between bleached and unbleached within time point 2, submissive behaviours occurred on average at a rate 174.0% higher in unbleached than in bleached anemones, but again this difference was not significant (ratio = 2.741, CI = 0.845–8.890, *z*-ratio = 1.679, *p*-value = 0.093).

There was no difference in submissive behaviours between rank 3 and rank 2 (emmeans contrast: ratio = 2.09, CI = 0.831–5.250, *z*-ratio = 1.873, *p*-value = 0.147) while both were significantly more submissive than rank 1 (rank 1–rank 2: ratio = 7.24, s.e. = 1.666–31.470, *z*-ratio = 3.157, *p*-value = 0.005; rank 1–rank 3: ratio = 15.12, CI = 3.163–72.280, *z*-ratio = 4.069, *p*-value = < 0.001). All other covariates had no significant impact on the rate of submissive behaviours (supplementary material, table S6.1). Fixed effects alone accounted for 19.4% of variance while fixed and random effects accounted for 22.0% of variance (marginal *R*^2^ = 0.194, conditional *R*^2^ = 0.220).

### 3.3. Mutualistic behaviours

#### 3.3.1. Maintenance

The rate of maintenance behaviours was not impacted by bleaching of the host or timepoint during the heatwave (figure 3D). Rate of maintenance behaviours displayed by anemonefish increased on average by 1.4% in unbleached anemones at time point 2 (emmeans contrast: ratio = 1.014, CI = 0.573–1.700, *z*-ratio = 0.052, *p*-value = 0.959) while decreasing by 3.3% in bleached anemones (ratio = 0.967, CI = 0.732–1.460, *z*-ratio = −0.192, *p*-value = 0.848). The difference between groups on bleached and unbleached anemones was not significant (time point 1: ratio = 1.253, CI = 0.648–2.420, *z*-ratio = 0.670, *p*-value = 0.503; time point 2: ratio = 1.314, CI = 0.685–2.520, *z*-ratio = 0.822, *p*-value = 0.411).

Rank 3 displayed maintenance behaviours at 35.1% less on average than rank 2 (emmeans contrast: ratio = 0.649, CI = 0.445–0.948, *z*-ratio = −2.677, *p*-value = 0.020) but there was no significant difference with rank 1 (ratio = 0.713, CI = 0.488–1.044, *z*-ratio = −2.078, *p*-value = 0.094), and no significant difference between rank 1 and rank 2 (ratio = 1.099, CI = 0.823–1.467, *z*-ratio = 0.763, *p*-value = 0.726). No other covariates had a significant impact on the rate of neutral behaviour (supplementary material, table S7.1). Fixed effects accounted for 1.5% of the variance while fixed and random effects accounted for 12.9% of the variance (marginal *R*^2^ = 0.015, conditional *R*^2^ = 0.129). This was the only model not significantly different from the null (supplementary material, table S9).

#### 3.3.2. Defence

The rate of defence behaviour exhibited by anemonefish increased in response to having a bleached host anemone (figure 3E). Rates of defence behaviour in bleached anemones increased significantly by 57.7% on average at time point 2 (emmeans contrast: ratio = 1.577, CI = 0.415–0.969, *z*-ratio = 2.104, *p*-value = 0.035), while those groups in unbleached anemones increased defence on average by 48.3% (ratio = 1.483, CI = 0.397–1.145, *z*-ratio = 1.460, *p*-value = 0.144). The difference between groups in bleached and unbleached anemones was not significant (time point 1: ratio = 1.383, CI = 0.691–2.770, *z*-ratio = 0.916, *p*-value = 0.360; time point 2: ratio = 1.300, CI = 0.652–2.590, *z*-ratio = 0.745, *p*-value = 0.456).

Rank 3 defended the anemone significantly less than both rank 1, by 37.3% (ratio = 0.627, CI = 0.456–0.862, *z*-ratio = −3.442, *p*-value = 0.002) and rank 2, by 33.0% (ratio = 0.670, CI = 0.485–0.924, *z*-ratio = −2.916, *p*-value = 0.010). But there was no difference in defence rates between ranks 1 and 2 (ratio = 0.936, CI = 0.745–1.176, *z*-ratio = −0.678, *p*-value = 0.777). No other covariates had a significant impact on the rates of defensive behaviour (supplementary material, table S8.1). Fixed effects accounted for 2.4% of variance while fixed and random effects accounted for 13.9% (marginal *R*^2^ = 0.024, conditional *R*^2^ = 0.139).

## 4. Discussion

Our results show that for habitat dependent marine species, such as *A. percula*, habitat degradation in the form of bleaching impacts behaviour more than thermal stress. Activity levels of anemonefish were consistent throughout the heatwave, independent of heat stress or bleaching. Bleaching primarily impacted which behaviour anemonefish performed while they were active. Social behaviours were impacted, with rate of aggressive behaviours increasing in bleached anemones even as temperatures cooled. On the other hand, mutualistic interactions with the anemone occurred at high rates in both bleached and unbleached anemones throughout the heatwave, with higher defence activity when anemones were bleached. Overall, our results indicate that in social animals dependent on mutualistic hosts, mutualistic interactions may be prioritized under environmental stress whereas social cohesion is likely to suffer.

Heat stress had no impact on the activity of anemonefish, remaining stable as temperature dropped throughout the heatwave. This is contrary to the effect found in other site attached damselfishes, where heat stress reduces swimming ability, triggered by reduction in aerobic scope [97], reduced muscle performance [98–100] or altered metabolic rate [67,75]. Habitat degradation from bleaching also had no significant impact on fish activity. By contrast, previous studies using experimentally induced bleaching, which could not measure the effect of heat stress, found anemonefish spending more time out of the anemone when it bleaches [75,76]. Reduced foraging rates in bleached anemones may explain the slightly greater decline in activity levels we found in bleached anemones once temperatures normalized [76]. Additionally, anemonefish in bleached anemones are more visible against the stark white background, making them more vulnerable to predation [54], and thus potentially more motivated to hide. Interestingly, fish which performed more behaviours per minute were less active overall (supplementary material, table S3.1). This suggests that fish may spend periods of time inactive, potentially hiding from predators, before undertaking a flurry of activity in which necessary behaviours are done. This may be why subordinate rank 3s, which are more vulnerable to predation, are also the most inactive throughout. Overall, while we find no significant evidence of heat stress or bleaching impacting fish activity, we highlight some trends which should be investigated further with a larger sample size.

We find that mutualistic behaviours remain a high priority for site attached fish during heat stress and during host bleaching. Mutualistic behaviours, which potentially benefit the anemone host [65], were consistently the most common anemonefish behaviours recorded, both at the height of the heatwave and as temperatures normalized. Fish in bleached anemones even increased their investment in defence behaviours as their habitat degraded. *A. percula* are obligate mutualists with *R. magnifica* and investment in the anemone may be prioritized even during times of high stress. This investment in defence may be prioritized, as venom production in bleached anemones decreases [101] and their ability to defend both themselves and the fish is impacted. Defence behaviours protect against anemone as well as fish predators [89], and maintenance behaviours potentially benefit the anemone through prevention of smothering and contributing to oxygenation [68,102]. Together, it is likely that these maintenance behaviours have a positive effect on anemone bleaching resistance and recovery [103], though more long-term behavioural studies and experiments are necessary to determine the fitness benefits of anemonefish behaviours for the anemone host.

Habitat degradation had a higher impact on social interactions in *A. percula* than heat stress. Aggression increased in bleached anemones despite the reduction in temperature from time point 1 to time point 2, while it remained consistent in unbleached anemones. Habitat or resource availability is a common driver of aggression throughout the animal kingdom (e.g. crayfish [104], lobster [105], birds [106,107]), and previous research on damselfish found increased levels of aggression in fish living among bleached corals [57,79,80]. Since anemonefish have no option to move host [108] competition for resources is heightened as the quality of the anemone declines along with food availability [109]. Like other damselfish and other fish species, we found that that the higher ranks exhibited higher rates of aggressive and lower rates of submissive behaviour [57,79,110]. There was no response in submissive behaviour to the increased levels of aggression displayed in bleached anemones but submission was consistently higher in unbleached anemones.

Neutral, or social, interactions are used to promote social group cohesion and in anemonefish are used to compare body size and maintain the strict size hierarchy [70,111,112]. Our results showed no difference in rates of neutral behaviour between fish on bleached and unbleached anemones, suggesting that this behaviour was unaffected by the environmental change experienced and potentially highlighting the importance of maintaining these behaviours. Across animal taxa, social behaviours are known to impact social group organization and convey information pertinent to social group cohesion (e.g. elephant fish [113]; dolphins [114]; primates [115–117]). For anemonefishes, maintaining high levels of social information in times of environmental stress may help mitigate aggressive actions by demonstrating the size based social order remains intact and thereby evictions are avoided [118]. While we found no significant differences in rate of neutral behaviour, we did see a trend of steeper decline in bleached anemones and this together with the high rates of aggression suggests that the hierarchy structure of social groups is threatened by habitat degradation [119–122]. Decreases in habitat quality have been shown to decrease sociality in many taxa, from cooperatively breeding birds [123] to large mammals such as polar bears and primates [124,125]. Environmental change causes breakdowns in social groups which can trigger extended periods of disturbance owing to group instability even after the initial environmental disturbance has past [126]. How long these behavioural changes last and the medium to long-term effects on group fitness are potential targets of future research.

## 5. Conclusions

Our study has highlighted and differentiated the effects of both heat stress and host degradation in an obligate mutualist. We show that the maintenance of the mutualistic anemone is not affected during periods of high stress and defence is prioritized even further, whereas social cohesion within groups declines, with potential fitness consequences in the near term. Our results highlight the importance of considering multiple aspects of environmental change and how they can have different yet simultaneous consequences. With human induced environmental change altering many ecosystems around the world, it is critical to understand how species use behavioural plasticity to mitigate environmental stress and habitat degradation.

## Supplementary Material



## Data Availability

The dataset supporting this article has been uploaded as part of the supplementary material. The code analysing the data supporting this article has been uploaded as part of the supplementary material [127]. All full model outputs supporting the article have been included as part of the supplementary material. All data and analysis code from this study are available to download at Zenodo [128].
