# Supplementary material for: Anemonefish prioritize mutualistic partner over social group during a marine heatwave
Source: R Soc Open Sci. 2026 Nov 11;13(11):261623. doi: 10.1098/rsos.261623 (PMC13750922; doi:10.1098/rsos.261623)
Supplement: Supplementary file 1 [file rsos.261623.zip › rsos.261623/rsos261623_si_003.docx]

**Anemonefish prioritise mutualistic partner over social group during marine heatwave – Supplementary Materials**

**Table 1. Ethogram used to score fish behaviour, modified from *Rueger et al. 2022* and Gaffney *et al.* 2026**

| **Behavioural Category** | **Sub-Category** | **Behaviour** | **Description** |
| --- | --- | --- | --- |
| Social | Aggressive | Chase - Aggressive | Fish swims rapidly towards conspecific and conspecific flees |
|  |  | Bites | Fish bites another fish |
|  |  | Jerk – Aggressive | Short, rapid and directed head-jerk movement towards conspecific (occurs in close proximity) |
|  |  | Flick – Aggressive | Rapidly darts and turns, flicking their caudal fin (occurs prior to or during aggressive activity) towards conspecific |
|  |  | Aggressive Display | Fish meets another, turns side on with erect fins, stiffened body posture |
|  | Neutral | Meeting | Fish simultaneously approach each other to within a body length (may be followed by a soft touch) |
|  |  | Soft Touch | Fish make contact with finds or body before separating again, preceded by meeting |
|  |  | Associate | Two fish with two body lengths of each other for at least two seconds |
|  |  | Approach | One fish swims towards another (with no rapid, direct bursts of speed) to within one body length (maybe followed by soft touch) |
|  |  | Follows | Fish follows another fish within 1 body length for at least a few seconds |
|  | Submissive | Flees | Fish rapidly swims away after receiving aggression from conspecific |
|  |  | Moving Away | Swims out of the way of a passing conspecific (not following aggression) |
|  |  | Body Shake | Fish shakes body side to side in a jolty fashion |
|  |  | S-Bend | Displays S-Bend posture |
|  |  | C-Bend | Displays C-Bend posture |
| Mutualistic | Maintenance | Bite Anemone | Fish bites mouth, tentacles or foot of anemone |
|  |  | Massage Anemone | Fish moves vigorously against anemone foot (often observed when anemone is contracting) |
|  |  | Cleaning | Fish picks up sand or debris and spits it outside the anemone |
|  |  | Aerate | Swimming vigorously through the anemone tentacles |
|  | Defence | Chase - Defence | Rapidly swimming towards predator/competitor, which flees |
|  |  | Guards | Body/head position following a predator/competitor, often moving up and down or side to side (mostly occurs around anemone territory) |
|  |  | Head Down Display | Face their head downwards, often move upwards (tail first_ in this position |
|  |  | Flick – Defence | Rapidly darts and turns, flicking their caudal fin (occurs prior to or during aggressive contact) |
|  |  | Jerk – Defence | Short, rapid and directed head-jerk movement towards heterospecific |
| Out of Sight | Out of Sight | Out of Sight | Fish is out of sight. Often below tentacles |

**Table 2. Neutral Rate Behaviour Model Sensitivity Test Output**

| **Predictor** | **β** | **95% CI** | **z** | **p** |
| --- | --- | --- | --- | --- |
| Time Point (TP2) | -0.184 | -0.888–0.520 | -0.51 | 0.609 |
| Bleaching Status (Bleached) | -0.278 | -1.492–0.937 | -0.45 | 0.654 |
| Group size | 0.226 | -0.738–1.190 | 0.46 | 0.646 |
| Anemone size | -0.363 | -0.955–0.229 | -1.20 | 0.230 |
| Subject rank: 2 | 0.283 | 0.047–0.519 | 2.35 | 0.019* |
| Subject rank: 3 | 0.216 | -0.076–0.507 | 1.45 | 0.147 |
| Microhabitat Temperature | 0.065 | -0.211–0.342 | 0.46 | 0.644 |
| Time Point × Bleaching Status | -0.096 | -0.580–0.388 | -0.39 | 0.697 |
| **Random effects** |  |  |  |  |
| Anemone ID | 1.4326 |  |  |  |
| **Model statistics** |  |  |  |  |
| N observations | 126 |  |  |  |
| N anemones | 23 |  |  |  |
| AIC | 607.9 |  |  |  |
| BIC | 639.1 |  |  |  |
| Dispersion | 0.98 |  |  |  |
| ΔAIC vs. full model | -2.0 |  |  |  |
| LRT p-value | 1.000 |  |  |  |

**Table 3.1. General Activity Model Output**

| **Predictor** | **β** | **95% CI** | **df** | **t** | **p** |
| --- | --- | --- | --- | --- | --- |
| Time Point (TP2) | -0.045 | -0.230–0.141 | 74.71 | -0.48 | 0.633 |
| Bleaching Status (Bleached) | -0.048 | -0.194–0.099 | 27.95 | -0.67 | 0.510 |
| Behavioural rate | -2.706 | -4.892–-0.519 | 88.74 | -2.46 | 0.016* |
| Subject rank: 2 | -0.042 | -0.132–0.047 | 34.89 | -0.96 | 0.344 |
| Subject rank: 3 | -0.248 | -0.347–-0.149 | 38.57 | -5.06 | <0.001*** |
| Group size | -0.054 | -0.187–0.080 | 47.47 | -0.81 | 0.424 |
| Anemone size | 0.021 | -0.041–0.083 | 21.42 | 0.70 | 0.493 |
| Microhabitat Temperature | -0.053 | -0.123–0.018 | 75.48 | -1.48 | 0.142 |
| Time Point × Bleaching Status | -0.027 | -0.174–0.119 | 59.78 | -0.38 | 0.709 |
| **Random effects** |  |  |  |  |  |
| Anemone ID | 0.0085 |  |  |  |  |
| Fish ID | 0.0072 |  |  |  |  |
| Residual | 0.0302 |  |  |  |  |
| **Model statistics** |  |  |  |  |  |
| Marginal R² | 0.254 |  |  |  |  |
| Conditional R² | 0.509 |  |  |  |  |
| N observations | 126 |  |  |  |  |
| N anemones | 23 |  |  |  |  |
| N Fish | 65 |  |  |  |  |
|  |  |  |  |  |  |

**Table 3.2. Emmeans contrast of time point 1 and time point 2 for fish in bleached and unbleached anemones**

| **Bleaching status** | **Contrast** | **Estimate** | **95% CI** | **df** | **t** | **p** |
| --- | --- | --- | --- | --- | --- | --- |
| Unbleached | TP1 − TP2 | 0.045 | -0.144–0.233 | 77.4 | 0.47 | 0.639 |
| Bleached | TP1 − TP2 | 0.072 | -0.057–0.201 | 78.2 | 1.11 | 0.269 |

**Table 3.3. Emmeans contrast of bleached and unbleached anemones for fish during time point 1 and time point 2**

| **Time Point** | **Contrast** | **Estimate** | **95% CI** | **df** | **t** | **p** |
| --- | --- | --- | --- | --- | --- | --- |
| TP1 | Unbleached − Bleached | -0.048 | -0.194–0.098 | 30.2 | -0.67 | 0.510 |
| TP2 | Unbleached − Bleached | -0.075 | -0.224–0.074 | 32.9 | -1.03 | 0.312 |

**Table 4.1. Rate of Aggressive Behaviours Model Output**

| **Predictor** | **β** | **95% CI** | **z** | **p** |
| --- | --- | --- | --- | --- |
| Time Point (TP2) | -0.939 | -2.803–0.924 | -0.99 | 0.323 |
| Bleaching Status (Bleached) | -0.111 | -1.935–1.712 | -0.12 | 0.905 |
| Group size | 0.514 | -0.528–1.555 | 0.97 | 0.334 |
| Anemone size | 0.120 | -0.618–0.857 | 0.32 | 0.751 |
| Subject rank: 2 | 0.024 | -0.496–0.545 | 0.09 | 0.928 |
| Subject rank: 3 | -1.121 | -1.912–-0.330 | -2.78 | 0.005** |
| Microhabitat Temperature | 0.337 | -0.139–0.814 | 1.39 | 0.165 |
| Time Point × Bleaching Status | 2.087 | 0.303–3.871 | 2.29 | 0.022* |
| **Random effects** |  |  |  |  |
| Anemone ID | 2.2839 |  |  |  |
| Fish ID | 0.0426 |  |  |  |
| **Model statistics** |  |  |  |  |
| N observations | 126 |  |  |  |
| N anemones | 23 |  |  |  |
| N fish | 65 |  |  |  |
| AIC | 316.5 |  |  |  |
| BIC | 350.5 |  |  |  |
| Dispersion | 1.57 |  |  |  |

**Table 4.2. Emmeans contrast of time point 1 and time point 2 for fish in bleached and unbleached anemones**

| **Bleaching status** | **Contrast** | **Ratio** | **95% CI** | **z** | **p** |
| --- | --- | --- | --- | --- | --- |
| Unbleached | TP2 / TP1 | 0.391 | 0.061 – 2.520 | -0.998 | 0.323 |
| Bleached | TP2 / TP1 | 3.150 | 1.465 – 6.770 | 2.937 | 0.003** |

**Table 4.3. Emmeans contrast of bleached and unbleached anemones for fish during time point 1 and time point 2**

| **Time Point** | **Contrast** | **Ratio** | **95% CI** | **z** | **p** |
| --- | --- | --- | --- | --- | --- |
| TP1 | Unbleached / Bleached | 1.118 | 0.180 – 6.920 | 0.120 | 0.905 |
| TP2 | Unbleached / Bleached | 0.139 | 0.016 – 1.240 | -1.767 | 0.077 |

**Table 5.1. Rate of neutral behaviours model output**

| **Predictor** | **β** | **95% CI** | **z** | **p** |
| --- | --- | --- | --- | --- |
| Time Point (TP2) | -0.184 | -0.888–0.520 | -0.51 | 0.609 |
| Bleaching Status (Bleached) | -0.278 | -1.492–0.937 | -0.45 | 0.654 |
| Group size | 0.226 | -0.738–1.190 | 0.46 | 0.646 |
| Anemone size | -0.363 | -0.955–0.229 | -1.20 | 0.230 |
| Subject rank: 2 | 0.283 | 0.047–0.519 | 2.35 | 0.019* |
| Subject rank: 3 | 0.216 | -0.076–0.507 | 1.45 | 0.147 |
| Microhabitat Temperature | 0.065 | -0.211–0.342 | 0.46 | 0.644 |
| Time Point × Bleaching Status | -0.096 | -0.580–0.388 | -0.39 | 0.697 |
| **Random effects** |  |  |  |  |
| Anemone ID | 1.4326 |  |  |  |
| Fish ID | 0.0000 |  |  |  |
| **Model statistics** |  |  |  |  |
| N observations | 126 |  |  |  |
| N anemones | 23 |  |  |  |
| N fish | 65 |  |  |  |
| AIC | 609.9 |  |  |  |
| BIC | 644.0 |  |  |  |
| Dispersion | 0.98 |  |  |  |

**Table 5.2. Emmeans contrast of time point 1 and time point 2 for fish in bleached and unbleached anemones**

| **Bleaching status** | **Contrast** | **Ratio** | **95% CI** | **z** | **p** |
| --- | --- | --- | --- | --- | --- |
| Unbleached | TP2 / TP1 | 0.832 | 0.411 – 1.680 | -0.512 | 0.609 |
| Bleached | TP2 / TP1 | 0.756 | 0.463 – 1.230 | -1.121 | 0.262 |

**Table 5.3. Emmeans contrast of bleached and unbleached anemones for fish during time point 1 and time point 2**

| **Time Point** | **Contrast** | **Ratio** | **95% CI** | **z** | **p** |
| --- | --- | --- | --- | --- | --- |
| TP1 | Unbleached / Bleached | 1.320 | 0.392–4.447 | 0.448 | 0.654 |
| TP2 | Unbleached / Bleached | 1.453 | 0.427–4.942 | 0.654 | 0.550 |

**Table 6.1. Rate of submissive behaviours model output**

| **Predictor** | **β** | **95% CI** | **z** | **p** |
| --- | --- | --- | --- | --- |
| Time Point (TP2) | -0.832 | -2.622–0.957 | -0.91 | 0.362 |
| Bleaching Status (Bleached) | -0.655 | -1.741–0.431 | -1.18 | 0.237 |
| Group size | -0.682 | -1.770–0.406 | -1.23 | 0.219 |
| Anemone size | -0.311 | -0.846–0.225 | -1.14 | 0.256 |
| Subject rank: 2 | 1.980 | 0.751–3.208 | 3.16 | 0.002** |
| Subject rank: 3 | 2.716 | 1.408–4.024 | 4.07 | <0.001*** |
| Microhabitat Temperature | -0.255 | -0.888–0.379 | -0.79 | 0.431 |
| Time Point × Bleaching Status | -0.354 | -1.870–1.163 | -0.46 | 0.648 |
| **Random effects** |  |  |  |  |
| Anemone ID | 0.2229 |  |  |  |
| Fish ID | 0.0000 |  |  |  |
| **Model statistics** |  |  |  |  |
| N observations | 126 |  |  |  |
| N anemones | 23 |  |  |  |
| N fish | 65 |  |  |  |
| AIC | 219.2 |  |  |  |
| BIC | 253.3 |  |  |  |
| Dispersion | 1.44 |  |  |  |

**Table 6.2. Emmeans contrast of time point 1 and time point 2 for fish in bleached and unbleached anemones**

| **Bleaching status** | **Contrast** | **Ratio** | **95% CI** | **z** | **p** |
| --- | --- | --- | --- | --- | --- |
| Unbleached | TP2 / TP1 | 0.435 | 0.073 – 2.600 | -0.911 | 0.362 |
| Bleached | TP2 / TP1 | 0.306 | 0.077 – 1.220 | -1.682 | 0.093 |

**Table 6.3. Emmeans contrast of bleached and unbleached anemones for fish during time point 1 and time point 2**

| **Time Point** | **Contrast** | **Ratio** | **95% CI** | **z** | **p** |
| --- | --- | --- | --- | --- | --- |
| TP1 | Unbleached / Bleached | 1.924 | 0.650–5.702 | 1.181 | 0.237 |
| TP2 | Unbleached / Bleached | 2.741 | 0.845–8.892 | 1.679 | 0.093 |

**Table 7.1. Rate of maintenance behaviour model output**

| **Predictor** | **β** | **95% CI** | **z** | **p** |
| --- | --- | --- | --- | --- |
| Time Point (TP2) | 0.014 | -0.528–0.557 | 0.05 | 0.959 |
| Bleaching Status (Bleached) | -0.225 | -0.885–0.434 | -0.67 | 0.503 |
| Group size | -0.204 | -0.702–0.293 | -0.80 | 0.421 |
| Anemone size | -0.044 | -0.327–0.239 | -0.30 | 0.762 |
| Subject rank: 2 | 0.094 | -0.148–0.336 | 0.76 | 0.445 |
| Subject rank: 3 | -0.337 | -0.656–-0.019 | -2.08 | 0.038* |
| Microhabitat Temperature | -0.041 | -0.238–0.157 | -0.40 | 0.686 |
| Time Point × Bleaching Status | -0.048 | -0.454–0.358 | -0.23 | 0.817 |
| **Random effects** |  |  |  |  |
| Anemone ID | 0.3620 |  |  |  |
| Fish ID | 0.0664 |  |  |  |
| **Model statistics** |  |  |  |  |
| N observations | 126 |  |  |  |
| N anemones | 23 |  |  |  |
| N fish | 65 |  |  |  |
| AIC | 942.0 |  |  |  |
| BIC | 976.0 |  |  |  |
| Dispersion | 3.76 |  |  |  |

**Table 7.2. Emmeans contrast of time point 1 and time point 2 for fish in bleached and unbleached anemones**

| **Bleaching status** | **Contrast** | **Ratio** | **95% CI** | **z** | **p** |
| --- | --- | --- | --- | --- | --- |
| Unbleached | TP2 / TP1 | 1.014 | 0.573 – 1.700 | 0.052 | 0.959 |
| Bleached | TP2 / TP1 | 0.967 | 0.732 – 1.460 | -0.192 | 0.848 |

**Table 7.3. Emmeans contrast of bleached and unbleached anemones for fish during time point 1 and time point 2**

| **Time Point** | **Contrast** | **Ratio** | **95% CI** | **z** | **p** |
| --- | --- | --- | --- | --- | --- |
| TP1 | Unbleached / Bleached | 1.253 | 0.648–2.422 | 0.670 | 0.503 |
| TP2 | Unbleached / Bleached | 1.314 | 0.685–2.523 | 0.822 | 0.411 |

**Table 8.1. Rate of defence behaviour model output**

| **Predictor** | **β** | **95% CI** | **z** | **p** |
| --- | --- | --- | --- | --- |
| Time Point (TP2) | 0.394 | -0.135–0.924 | 1.46 | 0.144 |
| Bleaching Status (Bleached) | -0.324 | -1.017–0.369 | -0.92 | 0.360 |
| Group size | 0.563 | -0.020–1.146 | 1.89 | 0.058 |
| Anemone size | -0.005 | -0.308–0.299 | -0.03 | 0.976 |
| Subject rank: 2 | -0.066 | -0.257–0.125 | -0.68 | 0.498 |
| Subject rank: 3 | -0.467 | -0.733–-0.201 | -3.44 | <0.001*** |
| Microhabitat Temperature | 0.187 | -0.056–0.429 | 1.51 | 0.131 |
| Time Point × Bleaching Status | 0.061 | -0.314–0.437 | 0.32 | 0.749 |
| **Random effects** |  |  |  |  |
| Anemone ID | 0.4459 |  |  |  |
| Fish ID | 0.0051 |  |  |  |
| **Model statistics** |  |  |  |  |
| N observations | 126 |  |  |  |
| N anemones | 23 |  |  |  |
| N fish | 65 |  |  |  |
| AIC | 938.8 |  |  |  |
| BIC | 972.8 |  |  |  |
| Dispersion | 3.46 |  |  |  |

**Table 8.2. Emmeans contrast of time point 1 and time point 2 for fish in bleached and unbleached anemones**

| **Bleaching status** | **Contrast** | **Ratio** | **95% CI** | **z** | **p** |
| --- | --- | --- | --- | --- | --- |
| Unbleached | TP2 / TP1 | 1.483 | 0.397 – 1.145 | 1.460 | 0.144 |
| Bleached | TP2 / TP1 | 1.577 | 0.415 – 0.969 | 2.104 | 0.035* |

**Table 8.3. Emmeans contrast of bleached and unbleached anemones for fish during time point 1 and time point 2**

| **Time Point** | **Contrast** | **Ratio** | **95% CI** | **z** | **p** |
| --- | --- | --- | --- | --- | --- |
| TP1 | Unbleached / Bleached | 1.383 | 0.691–2.765 | 0.916 | 0.360 |
| TP2 | Unbleached / Bleached | 1.300 | 0.652–2.595 | 0.745 | 0.456 |

**Table 9. Likelihood-ratio tests comparing full and null models of behaviour category models**

| **Behaviour** | **χ²** | **df** | **p** |
| --- | --- | --- | --- |
| Aggressive | 19.88 | 8 | 0.011* |
| Neutral | 17.87 | 8 | 0.022* |
| Submissive | 28.13 | 8 | <0.001*** |
| Maintenance | 9.73 | 8 | 0.285 |
| Defence | 19.40 | 8 | 0.013* |
